# The community counts: a participatory approach to social audits

**DOI:** 10.1186/1472-6963-11-S2-I1

**Published:** 2011-12-21

**Authors:** Susanna Hausmann-Muela

**Affiliations:** 1 Centro de Investigación de Enfermedades Tropicales (CIET), Universidad Autónoma de Guerrero, Calle Pino, El Roble, Acapulco, México

## 

“The people have to be seen … as actively involved—given the opportunity—in shaping their own destiny, and not just as passive recipients of the fruits of cunning development programs”

*Amartya Sen*, *Development as Freedom *[1]

Community matters – community responds. Yet many health planners still consider people as passive recipients of programmes.

WHO Director-General Dr. Margaret Chan drew attention to this in the global approach to the H1N1 pandemic: “Although the virus has not yet delivered any devastating surprises, we have seen some surprises on other fronts. We anticipated problems in producing enough vaccine fast enough, and this did indeed happen. But we did not anticipate that people would decide not to be vaccinated” [2].

Social audits respond to the voice of different stakeholders, including intended beneficiaries, in order to improve health planning and service delivery. Over the last two decades, government and private donors have increased their demands for evidence of the impacts of their supported programmes, to enhance accountability and transparency of investments. However, most decisions in health care are still taken without benefit of solid evidence from the concerned communities and intended beneficiaries. While it is true that people are increasingly consulted, their voice is still a faint murmur at policy and planning levels. The CIET approach to social audit responds to this long standing shortfall.

This Supplement marks CIET's 25 years of experience in developing and conducting social audits. Over this period, the methodology has evolved, from the early sentinel community surveillance and community-based service-delivery surveys, through reiterative survey and feedback cycles that propose service changes and monitor their effects, to randomised controlled trials - that measure the impact of interventions. The systematic and rigorous social audit methods provide solid evidence *from* communities that encourage health planners to plan *with* and *for* the communities.

While the methodological approach has evolved and matured over the past quarter century, with social audits covering a wide range of topics in many countries, the underlying philosophy of CIET's social audits has endured.

CIET's social audit approach was inspired by the Italian labour movement and its “alternativa operaia” [3] that involved workers and their knowledge in the conduct of occupational epidemiology. Applied in Nicaragua and Honduras in the context of monitoring the “child survival revolution” in the mid-1980s [4], this approach found common ground with the Latin American tradition of participatory action research, theoretically grounded in Freire's philosophy of education. Social audits follow a dialogic approach to health-for-all, based on a two-way, symmetrical communication between professionals and the social groups that health interventions are aimed at. Using Freire's [5] words, “[i]t is not our role to speak to the people about our own view of the world, nor to attempt to impose that view on them, but rather to dialogue with the people about their view and ours” (p.96).

A key feature of CIET's social audits, as distinct from Freire's approach, is the use of epidemiology in gathering and systematizing evidence from communities. The intention is to combine scientific rigour with community engagement and social commitment. A methodology to deal with people's live-worlds (see Nations [6]) should be rigorous but not rigid (the rigor mortis of a blinkered science). As Neil Andersson [7] states in the introductory paper of this Supplement: “behind our social audit approach is the idea of epidemiology as an evolving and self-organising system, a language rather than a rigid tool, with increasingly informed community engagement and increasing relevance of the emerging solutions”.

CIET social audits systematically share reliable, scientifically valid and actionable local evidence with communities and service providers, through mechanisms including focus groups and dialogues with community and service representatives. This formalised mechanism for community participation in interpreting local evidence and developing solutions [8] nourishes the critical voice issuing from this dialogue. This process of socializing of evidence for participatory action (SEPA) is iterative: repeated cycles of research and action building confidence among communities to identify and solve their own problems.

The introductory and concluding papers of this supplement discuss the evolution of CIET's social audit methods, the lessons learned, and the way forward to the next generation of social audits. Other papers illustrate the spread of social audits in the sphere of health: across five continents and covering topics from corruption in medical education in Mexico, through local health interventions in indigenous communities in Canada, to national services reform programmes in South Asia. Nearly all the social audits described in this supplement had at least two cycles of data collection, analysis and feedback, illustrating the opportunities and challenges of an evidence-based dialogue with different stakeholders. The early micro-regional planning initiative in Mexico describes how social audit spurred a long-term dialogue among communities and with local governments. Some of the repeat cycles showed improvements following actions guided by the initial cycle, as in the Mexico medical school audit, in anti-corruption efforts in Nicaragua, and in reproductive health services in the Maldives.

In other cases, such as in Bangladesh, Pakistan and South Africa, repeat social audits provided evidence reform programmes and interventions were having little or no impact on the experience and views of communities and service users. Several of the papers describe social audits conducted in the previous century and reflect on their challenges and lessons, while others describe new social audit programmes, such as in Nigeria and the Northwest Territories of Canada. Three papers make specifically methodological contributions: one draws on experience of collecting evidence about unofficial payments in six countries; another describes the use of maps for communicating social audit findings; and another describes a method of taking account of clustering in the cross-sectional surveys which are part of most social audits.

CIET's use of epidemiology to build the voice of the communities into planning has been successful in many cases. In an era where the search for technological rather than human solutions predominates, and where impact is measured as 'social returns on investments', it is encouraging to see robust and field-tested methods for building social capital and community empowerment.

There are other examples of epidemiological approaches to building the community voice into planning. In Latin America the tradition of participatory action research (PAR) is rooted in the work of Paulo Freire [5] and Orlando Fals Borda [9] In the UK and the Commonwealth countries rapid rural appraisal associated with Robert Chambers shares much of the same philosophy [10]. In North America such approaches are generally referred to as community-based participatory research (CBPR) [11]. In Nepal, Manandhar and colleagues have been conducting studies that have incorporated social mobilisation into cluster randomised controlled trials involving maternal and child health [12,13].

Parallel movements of participatory action research are gaining strength in public health and transdisciplinary research; formative research [14] and asset-based community development [15] are well developed and have good track-records in implementation and development programs.

Common to these approaches and the CIET social audit approach is the recognition that behaviour change and community empowerment take time. Sustainable change is a long-term social process; there is no short-cut and no miraculous technological quick-fix to show immediate results. A systematic review of CBPR studies in 2004 found many studies that had strong community-institution collaborations while relatively few combined such collaboration with solid research methods [16]. The research methods presented in this supplement are both solid and transparent.

This Supplement on CIET social audit advocates for a participatory approach in health policy and planning, that gives people the opportunity to participate in shaping their own destiny – that gives them a voice.
